# Identification of New Epididymal Luminal Fluid Proteins Involved in Sperm Maturation in Infertile Rats Treated by Dutasteride Using iTRAQ

**DOI:** 10.3390/molecules21050602

**Published:** 2016-05-11

**Authors:** Shu-Wu Xie, Guo-Ting Li, Li-Juan Qu, Yang Cao, Qian Wang, Jie-Yun Zhou, Rui-Hua Zhong, Xiang-Jie Guo, Yan Zhu

**Affiliations:** 1 Department of Reproductive Pharmacology, Key Lab of Reproduction Regulation of NPFPC, SIPPR, IRD, Fudan University, Shanghai 200032, China; xieshw@fudan.edu.cn (S.-W.X.); ligt89@sippr.org (G.-T.L.); zjy_sans@sippr.org (J.-Y.Z.); zrh8804@126.com (R.-H.Z.); gxj2009@sippr.org (X.-J.G.); 2 Department of Laboratory Medicine, Shanghai Eighth People’s Hospital, Shanghai 200233, China; 3 Yueyang Hospital of Integrated Traditional Chinese and Western Medicine, Shanghai 200437, China; cindy8813966@126.com; 4 Ming Qi Natural Healthcare Center, New York, NY 10016, USA; blackeyes614@hotmail.com

**Keywords:** dutasteride, rat epididymis, luminal fluids, iTRAQ, proteomics, drug targets, contraceptives, infertility therapy

## Abstract

*Background*: Spermatozoa become mature and acquire fertilizing capacity during their passage through the epididymal lumen. In this study, we identified new epididymal luminal fluid proteins involved in sperm maturation in infertile rats by dutasteride, a dual 5α-reductase inhibitor, in order to provide potential epididymal targets for new contraceptives and infertility treatment. *Methods*: Male rats were treated with dutasteride for 28 consecutive days. We observed the protein expression profiles in the epididymal luminal fluids in infertile and normal rats using isobaric tags for relative and absolute quantitation (iTRAQ) technique. The confidence of proteome data was validated by enzyme-linked immunosorbent assays. *Results*: 1045 proteins were tested, and 23 of them presented different expression profiling in the infertile and normal rats. The seven proteins were down-regulated, and 16 proteins were up-regulated. Among the seven proteins which were significantly down-regulated by dutasteride in the epididymal luminal fluids, there were three β-defensins (Defb2, Defb18 and Defb39), which maybe the key proteins involved in epididymal sperm maturation and male fertility. *Conclusions*: We report for the first time that dutasteride influences the protein expression profiling in the epididymal luminal fluids of rats, and this result provides some new epididymal targets for male contraception and infertility therapy.

## 1. Introduction

Testicular spermatozoa released into the seminiferous tubule are not yet mature or fertile. They become mature and acquire fertilizing capacity during the passage through the epididymal lumen [1,2]. In the course of the process of sperm maturation in the epididymal lumen, multiple changes occur in the sperm, including changes in morphology, biochemistry, physiology and the acquisition of fertilizing ability due to the interaction of epididymal secretory proteins with the spermatozoa [3,4,5]. The epididymal microenvironment surrounding the spermatozoa is maintained by active secretion and reabsorption throughout the tract and by the presence of a continuous blood barrier formed by tight junctions from the epithelial cells in the epididymis [6]. This barrier isolates the spermatozoa from the body fluids, from their formation to their maturation [7]. This physical separation by the blood barrier plays a vital role in protecting spermatozoa from autoimmune reactions that could occur due to specific epididymal protein secretions associated with the terminal cellular differentiation of the gametes [8,9].

The epididymal structure and functions are highly dependent on the presence of androgens [9]. Several previous studies have shown that dihydrotestosterone (DHT), but not testosterone (T), was the major androgen acting in the epididymis [10,11,12]. Therefore, the inhibition of DHT production is a suitable approach for studying the regulation of epididymal function and sperm maturation. Some studies have shown that dutasteride or its analogues can decrease DHT-dependent tissue weights (including epididymis) *in vivo* [13]. In some previous studies, dutasteride or its analogues had a drastic effect on epididymal gene expression in adult male rats [14]. However, some of the more dramatically affected genes are potentially involved in the formation of an optimal luminal microenvironment required for proper sperm maturation [11,15]. From our previous study, dutasteride could inhibit male rat fertility through interfering epididymal tight junctions, which could influence blood-epididymis barrier and change the composition of epididymal luminal fluids [16]. Thus, the changes in gene expression may impact epididymal luminal fluid protein constituents and consequently affect sperm maturation and male fertility.

From the above findings, it is deduced that dutasteride affects the epididymal function and impact male fertility by causing changes in the epididymal milieu, in which the spermatozoa mature, and not the spermatozoa. Investigation of protein expression changes in the epididymal luminal fluids after dutasteride treatment also supports the above facts, and this may indicate new epididymal targets for drug discovery of male fertility.

Proteomics research, an experimental approach widely used in the pharmacological research, has facilitated the analysis of the molecular mechanisms of drug action at the protein level. Serum protein expression profiles for different groups can be constructed, and differentially expressed proteins can be identified using two-dimensional (2D) electrophoresis, mass spectrometry (MS), and other proteomic methods including iTRAQ [17,18]. This study was designed to achieve the following goals: (1) to evaluate the effects of an altered androgen environment on protein expression profilings in cauda epididymal luminal fluids caused by dutasteride; (2) to use iTRAQ technology to further identified the screened differentially expressed protein profilings in cauda epididymal luminal fluids between normal and infertile rats treated by dutasteride.

## 2. Results

### 2.1. Effects of Dutasteride Treatment on Sperm Motility

**Figure 1:**
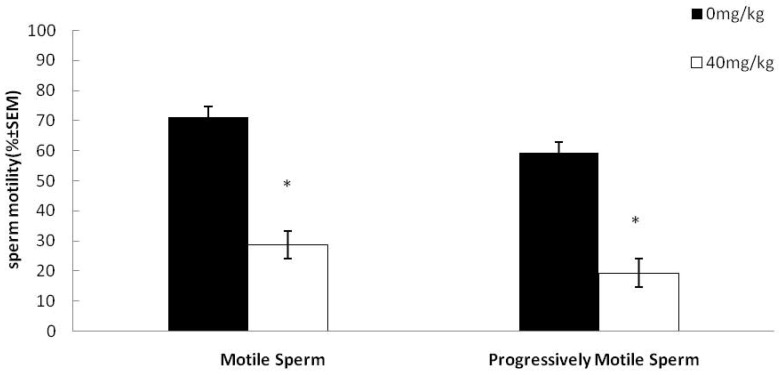
Effect of dutasteride treatment on sperm motility (**left**) and progressive motility (**right**). Data are represented as mean ± SEM. * *p* < 0.05 *versus* the control group, *n* = 8.

The sperm concentration did not significantly decrease in the dutasteride-treated group as compared to the control group. However, there was a significant decrease in the percentage of motile and progressively motile sperm (39% and 45%, respectively) from the cauda epididymis of treated male rats compared with untreated animals (Figure 1). Furthermore, dutasteride treatment also caused a significant decrease in other sperm motility parameters (Table 1).

### 2.2. Effects of Dutasteride Treatment on Sperm Morphology

**Figure 2:**
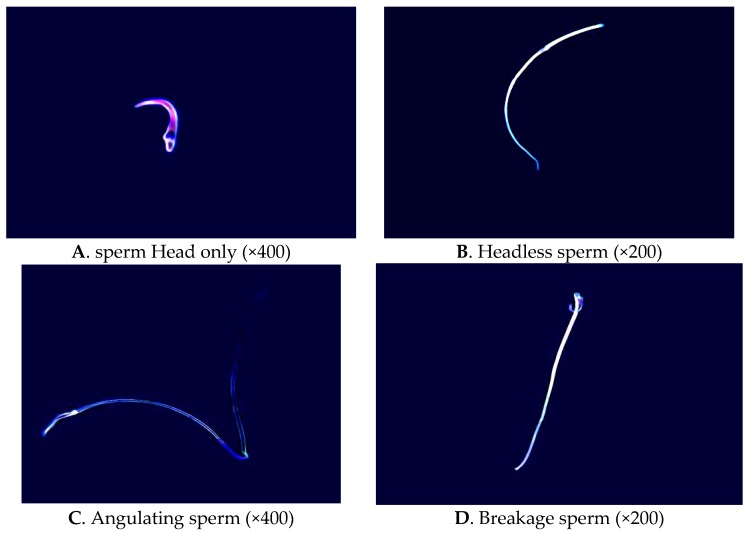
The structure of rat epididymal teratosperm under phase contrast microscope. (**A**) sperm Head only; (**B**) Headless sperm; (**C**) Angulating sperm; (**D**) Breakage sperm.

**Figure 3:**
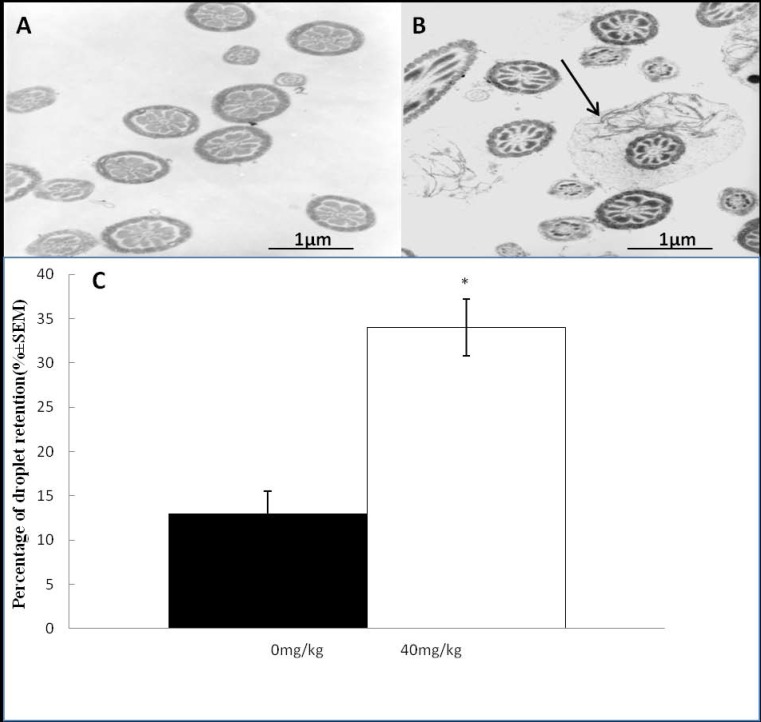
Electron microscopic analysis of the effect of dutasteride treatment on sperm morphology. (**A**) In the control group, there was no mid-piece sperm surrounded by cytoplasmic droplets; (**B**) After dutasteride treatment, there were some mid-piece sperms surrounded by cytoplasmic droplets (black arrows); (**C**) Comparison of the percentage of sperms droplet retention between the control and dutasteride groups. * *p* < 0.05 *versus* the control group.

As expected under phase-contrast microscopy observation, the number of abnormal sperm from the cauda epididymis of treated males was higher than that in the controls. The percentage of tailless, headless, broken, angulated and other abnormal sperm increased significantly with dutasteride treatment (Table 2, Figure 2). Analysis of the ultrastructure of sperm from the cauda epididymis revealed a significant increase in the percentage of mid-piece sperm that were surrounded by cytoplasmic droplets after dutasteride treatment (Figure 3).

### 2.3. Effects of Dutasteride Treatment on Male Mating and Fertility

In this study, although all of the males successfully mated with at least one virgin female in proestrus, there was a remarkable difference in the reproduction indices between the control and dutasteride-treated groups. All of the females that mated with the untreated males were pregnant; in contrast, there was a drastically significant decrease in the pregnancy rate for those females paired with the treated males (Table 3).

### 2.4. Effects of Dutasteride Treatment on Reproductive Organ Weights

**Figure 4:**
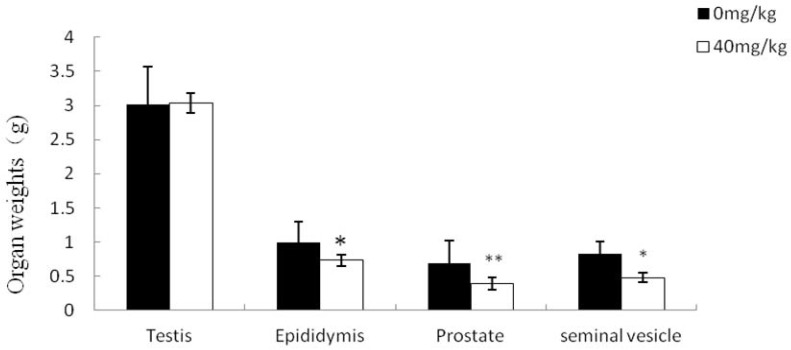
Effect of dutasteride treatment on reproductive organ weights. Data are represented as mean ± SEM. * *p* < 0.05 *versus* the control group; ** *p* < 0.01 *versus* the control group.

As expected, the weight of the testis, a T-dependent organ, was not affected in either group. However, there were remarkable decreases in the weight of the DHT-dependent tissues such as epididymis, seminal vesicle or prostate in the dutasteride-treated group (Figure 4).

### 2.5. Effects of Dutasteride Treatment on Serumal T and DHT

**Figure 5:**
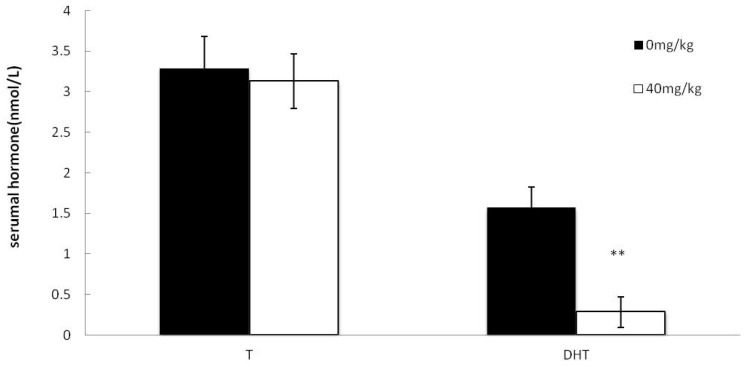
Effect of dutasteride treatment on serum T and DHT levels. Data are represented as mean ± SEM. ** *p* < 0.01 *versus* the control group, *n* = 8.

In this study, the serum T level was not affected by dutasteride treatment, but serum DHT level was significantly decreased in the dutasteride-treated group (Figure 5).

### 2.6. Differentially Expressed Proteins in Epididymal Luminal Fluids

1045 proteins were identified from 4239 unique peptides using the iTRAQ technique in this study. The 23 epididymal luminal fluid proteins were significantly differentially expressed between the dutasteride-treated group and the control group (>1.50 or <0.67). Seven of these proteins were down-regulated, and the other 16 proteins were up-regulated (Table 4).

**Figure 6:**
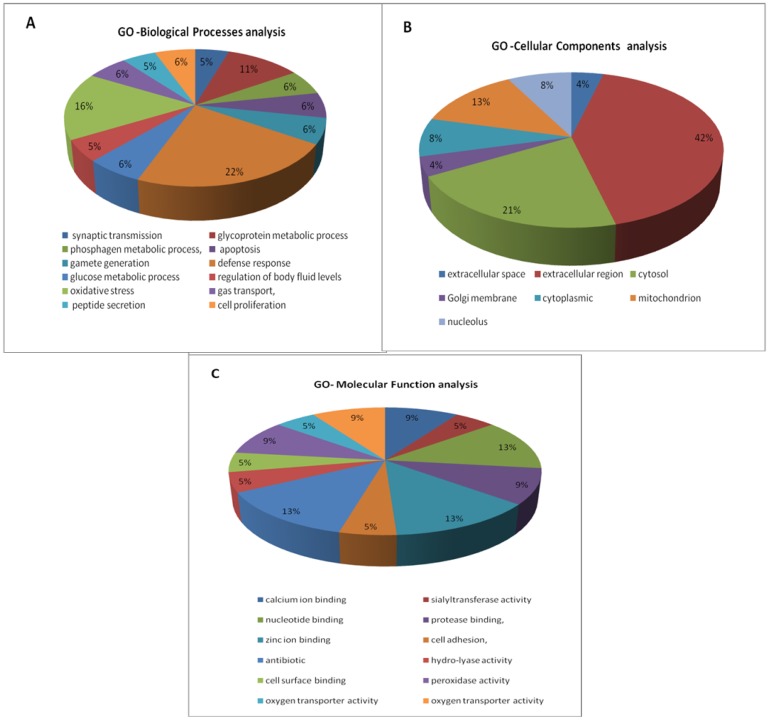
Pie diagrams of the proportion of differentially expressed epididymal proteins categorized by function between the dutasteride group and control group. (**A**) Gene Ontology analysis for biological processes; (**B**) Gene Ontology analysis for cellular components; (**C**) Gene Ontology analysis for molecular function.

Gene Ontology analysis for biological processes revealed that the main functional categories of these proteins were defense response (22%), oxidative stress (16%) and glycoprotein metabolic process (11%) (Figure 6A). According to Gene Ontology classification, most of these proteins are located in the extracellular region (Figure 6B). Gene Ontology analysis for molecular functions indicated that the major actions of these proteins were antibiotic (13%), calcium ion binding (13%) and nucleotide binding (13%). (Figure 6C).

Among the seven proteins that were significantly down-regulated by dutasteride in the epididymal luminal fluids, there were three β-defensins (Defb2, Defb18 and Defb39) that may be involved in sperm maturation and male fertility [9,19,20,21]. As for the 16 up-regulated proteins, there were eight proteins participating in material and energy metabolism, such as St6galnac2 and Ckmt1b, which could influence sperm motility and maturation when passed through the epididymal lumen. Among the other eight up-regulated proteins, S100b, S100a11, and Crip1 were involved in ion transportation, bringing about disorder of epididymal microenvironment. Gpx5 and Prdx6 play an important role in participating in oxidative stress, leading to epididymal sperm plasma membrane damage.

### 2.7. ELISA Detection of Selected Protein Concentrations

**Figure 7:**
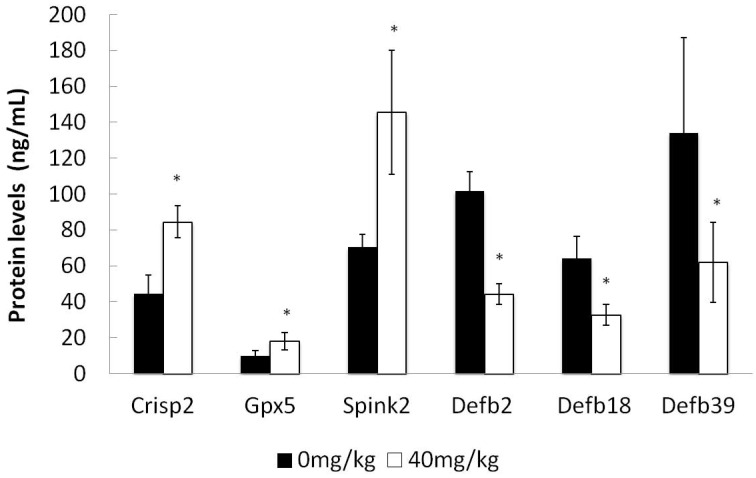
Analysis of proteins concentration data between dutasteride-treated group and control group by ELISA in epididymal luminal fluids. * *p* < 0.05 *versus* the control group.

We performed ELISA for six proteins including three up-regulated proteins (Crisp2, Gpx5, Spink2) and three down-regulated proteins (Defb2, Defb18, Defb39) to validate the alteration in epididymal luminal fluid protein expressions detected by iTRAQ. The results of ELISA measurement for these selected proteins were consistent with the iTRAQ data, although the changes in the concentration level were not equal to the iTRAQ results (Figure 7 and Table 4).

## 3. Discussion

In the present study, firstly, we established a male rat infertility model using dutasteride administration (40 mg/kg/d, po, 28 days) and evaluated the sperm motility and morphology, mating index, fertility index and pregnancy index. The results showed that dutasteride did not negatively affect sexual ability; thus, it is considered a new possible method for male contraception. Secondly, we discovered that the down-regulated epididymal luminal fluid proteins were three β-defensins, which were deemed to be related to epididymal sperm maturation/fertilization and were presumed to be the potential targets for new contraceptives. Thirdly, we also identified and analyzed the up-regulated epididymal luminal fluid proteins that are associated with material and energy metabolism and may be novel sites of action by dutasteride and other new male antifertility drugs. These differently expressed proteins may also be the conceivable targets for male infertility drug treatment.

It is well known that the sperm maturational changes in the epididymis result from sequential interactions with the changes in the milieu, particularly with the proteins secreted by the epididymal epithelium. Many proteins involved in sperm maturation have been identified as androgen-regulated proteins including β-defensins. Several β-defensins have been reported to be regulated by androgen in different species [9,22,23]. The results of our analysis showed that dutasteride could markedly inhibit the rat serumal DHT levels and down-regulate three β-defensins in an epididymal fluid, which are Defb2, Defb18 and Defb39. Several previous studies have suggested that β-defensins were essential for sperm motility [9,19,20,24,25]. The underlying mechanism may be that β-defensins can combine their antimicrobial activity with the ability to interact with sperm membrane receptors and modulate ion transport [24]. The three β-defensins in fluids may be major antifertility targets by dutasteride. These findings have shown that dutasteride also could inhibit expression of some proteins associated with material and energy metabolism in epididymal fluids, which possibly blocked sperm energy metabolism and function when they passed through the epididymal lumen. Consequently, sperm maturation was affected, which induced dysfunction in the sperm-egg fusion and male fertility finally.

Androgens affect fluid balance in the epididymis by regulating anion transport across the epithelium and modulate the epididymal expression of specific channels that mediate water transport in many tissues [21]. From our iTRAQ results, dutasteride could significantly up-regulate the expression of proteins such as S100b, S100a11, and Crip1 which are associated with anion transport in epididymal fluids, possibly resulting in metal ion binding and transportation disorders in the epididymal microenvironment. Gpx5 and Prdx6 were secreted from the epididymal epithelia; they protect cells from oxidative stress-induced lipid peroxidation and DNA mutation under the helps of the sperm plasma membrane [26]. Our iTRAQ and ELISA results suggested that dutasteride could alter the expression of Gpx5 and Prdx6 in the fluids. These altered expression levels indicate that dutasteride could induce oxidative challenge to sperm in epididymal fluids. The above androgen dependent proteins may have the different sensitivity and reactions to T or DHT. So the protein expression changes may have lagged behind the decreased androgen levels and it maybe also cause temporary markedly negative feedback [27]. With the further administration time prolonging and androgen levels decreasing, the androgen dependent protein expression levels would reduce accordingly [28,29]. The other up-regulated proteins, such as St6galnac2 and Ckmt1b, are associated with protein amino acid glycosylation and phosphocreatine metabolism. The alterations of these proteins expression may result in abnormal energy metabolism of epididymal sperm, accompanied by abnormal changes in sperm motility and morphology.

The findings of the present study suggest that molecular markers of male fertility are associated with both epididymal sperm and luminal fluid proteins. Although a few new epididymal luminal proteins were identified in this study, their origin and role in sperm maturation were not known and thus, there is a need for further investigation. In our next study, we hope to examine the caput, corpus and initial epididymal luminal fluids protein differences caused by treatment with dutasteride, and possibly explain the four types of epididymal luminal fluids protein expressions and their roles in the epididymal sperm maturation and fertility.

## 4. Materials and Methods

### 4.1. Animals and Treatment Procedure

Sixteen three-months-old Sprague-Dawley rats were obtained from Sino-British Sippr/BK Lab Animal Co. Ltd. (Shanghai, China), maintained under controlled light (12L:12D) and temperature (23 °C), and provided with food and water ad libitum. Male rats (330–350 g) were randomly divided into two groups (eight rats in each group) and gavaged with 1 mL/kg of 0 (control) or 40 mg/kg (treated) dutasteride (Gedian Humanwell Pharmaceutical Co., Ltd. Hubei, China) suspended in a 0.5% methylcellulose solution containing 0.2% Tween 20 for 28 consecutive days. Virgin female rats (220–250 g) were used for the mating study. Animals were anesthetized and sacrificed, and blood was collected from the abdominal aorta for T and DHT assays; then the testes, seminal vesicles, ventral prostates and epididymides were weighed. The eight left epididymides each group were prepared for epididymal luminal fluid proteomic analysis, and the eight right epididymides were used for analysis of sperm morphology and motility. The methods are described in our previous study [30]. All experimental studies were approved by the Animal Ethics Committee of Shanghai Institute of Planned Parenthood Research and complied with Regulations on the Care and Use of Laboratory Animals promulgated by The Ministry of Science and Technology of China.

### 4.2. Epididymal Luminal Fluids Collection

Epididymal luminal fluid samples were obtained from the eight left cauda epididymides each group by placing the epididymal tissue in PBS containing protease inhibitor cocktail and gently mincing the tissue with fine scissors. The resulting suspensions were centrifuged at 800× *g* (4 °C, 5 min) to remove tissues fragments. The supernatants were then recentrifuged at 12,000× *g* (4 °C, 15 min) to remove the sperm cells. The final supernatants were sperm-free luminal fluid fractions for protein extraction. In order to reduce the individual differences in two groups, we pooled the eight epididymal luminal fluid samples each group in an equal amount before proteomic analysis. Sample protein concentrations were determined by BCA protein assay (No. 23225, Pierce, Rockford, IL, USA).

### 4.3. Peptide Quantitation and iTRAQ Label

The epididymal luminal fluid samples were preconditioned using the PD-10 extraction desalting reagent (GE Healthcare, Bucks, UK and the eluent of each protein sample was quantitated using the Bradford method (Bio-Rad, CA, USA). Then, 20 mL of dissolution buffer, 1 mL of denaturant, and 2 mL of reducing agent (NP004, Thermo Fisher, San Jose, CA, USA,) were added to each tube, which contained 100 μg of the sample, before incubating at 60 °C for 1 h. Then, 1 mL cysteine blocking reagent and 10 mL of the trypsin solution were added to each tube. The tubes were then incubated at 37 °C overnight. Peptide mixtures were labeled using the 4-plex iTRAQ labeling kit (Applied Biosystems, Foster City, CA, USA) according to the iTRAQ Reagent Protocol. iTRAQ reagents 114 and 115 were used to label the peptides from the control group and the dutasteride group, respectively. After incubating the tubes at room temperature for 1 h, all of the iTRAQ-labeled tryptic peptide mixtures were combined to further purify [31].

### 4.4. Peptide Fractionation with Strong Cation Exchange (SCX) Chromatography

The fluid samples were fractionated by 2D liquid phase chromatography fractionation. Some of the fractions were combined according to the chromatogram. Two-dimensional chromatography was used to utilize the nano LC-Ultra 2D System (Eksigent, CA, USA).

### 4.5. LC-ESI -MS/MS Analysis by Q Exactive

Experiments were performed on a Q Exactive mass spectrometer that was coupled to Easy nLC (Thermo Fisher Scientific). 10 μL of each fraction was injected for nano LC-MS/MS analysis. The peptide mixture (5 μg) was loaded onto a C18-reversed phase column (Thermo Scientific Easy Column, 10 cm long, 75 μm inner diameter, 3 μm resin) in buffer A (0.1% Formic acid) and separated with a linear gradient of buffer B (80% acetonitrile and 0.1% Formic acid) at a flow rate of 250 nL/min controlled by IntelliFlow technology over 140 min. MS data were acquired using a data-dependent top10 method, dynamically choosing the most abundant precursor ions from the survey scan (300–1800 *m/z*) for HCD fragmentation. Determination of the target value is based on predictive Automatic Gain Control (pAGC). Dynamic exclusion duration was 60 s. Survey scans were acquired at a resolution of 70,000 at *m/z* 200 and resolution for HCD spectra were set to 17,500 at *m/z* 200. Normalized collision energy was 30 eV and the underfill ratio, which specifies the minimum percentage of the target value likely to be reached at maximum filling time, was defined as 0.1%. The instrument was run with peptide recognition mode enabled.

### 4.6. Sequence Database Searching and Quantitative Analysis

MS/MS spectra were searched using a MASCOT engine (version 2.2, Matrix Science, London, UK) embedded into Proteome Discoverer 1.3 (Thermo Electron, Waltham, MA, USA) against Uniprot_rat.fasta database (41,766 sequences, download at 8 March 2013) and the decoy database. For protein identification, the following options were used. Peptide mass tolerance = 20 ppm, MS/MS tolerance = 0.1 Da, Enzyme = Trypsin, Missed cleavage = 2, Fixed modification: Carbamidomethyl (C), iTRAQ4plex (K), iTRAQ4plex (N-term), Variable modification: Oxidation (M), FDR ≤ 0.01. The peptide fractionation ion peak strength values were quantitatively analyzed using Proteome Discoverer1.4 software (Thermo Fisher Scientific, Waltham, MA, USA).

### 4.7. Validation by Enzyme-Linked Immunosorbent Assay

To confirm the iTRAQ results, enzyme-linked immunosorbent assays (ELISA) were performed on the six detected epididymal luminal fluids proteins including 3 up-regulated proteins (Crisp2, Gpx5, Spink2) and three down-regulated proteins(Defb2, Defb18, Defb39), according to the manufacturer’s instructions (Cusabio Biotech Co. Ltd., Wuhan, China).

### 4.8. Data Analysis

For the iTRAQ analysis, we adopted the fold changes to compare the proteins expression differences between control and dutasteride group. A *t*-test was used to analyze the *p*-value of the value of log 2 (114/115). The protein with the fold changes cutoff ratio > 1.50 or < 0.67 as well as the *p* value less than 0.05 was designated the protein expression differences between two groups. The cellular component and biological process classifications of the selected proteins were annotated using the BLAST2GO software (version 3.0) with default settings [32]. GO functional classification and enrichment analysis were performed to identify GO terms that were significantly enriched in differentially expressed proteins between two groups using DAVID analysis [33].

## 5. Conclusions

We employed dutasteride treatment and iTRAQ technology to examine the effect of dutasteride on protein expression profiling in the epididymal luminal fluids. We preliminarily identified a proteomic profile of protein expression in the epididymal luminal fluids of rats with dutasteride-induced infertility, and this analysis potentially provides some new epididymal targets for male contraception and infertility investigations.

## Figures and Tables

**Table 1:** Effect of dutasteride treatment on other sperm motion parameters between the two groups (mean ± SEM).

Dutasteride	*n*	VAP (μm/s)	VSL (μm/s)	VCL (μm/s)	ALH (μm)	BCF (Hz)	STR (%)	LIN (%)
0 mg/kg	8	148.5 ± 23.0	108.3 ± 15.2	234.2 ± 33.0	14.8 ± 1.3	26.5 ± 2.6	72.3 ± 4.3	48.2 ± 3.6
40 mg/kg	8	135.8 ± 10.2	80.663 ± 7.2 **	233.5 ± 32.3	12.1 ± 1.6 *	21.3 ± 1.5	59.7 ± 5.2 **	36.1 ± 3.3 *

* *p* < 0.05 and ** *p* < 0.01 versus the control group. Average path velocity (VAP), Curvilinear velocity (VCL), Straight-line velocity (VSL), Amplitude of lateral head displacement (ALH), Beat cross frequency (BCF), Straightness (STR), Linearity (LIN).

**Table 2:** Comparison of percentage abnormal cauda epididymis sperm (mean ± SEM) between the dutasteride-treated and control groups.

Dutasteride	*n*	Headless (%)	Tailless (%)	Angulated (%)	Broken (%)	Other (%)	Total (%)
0 mg/kg	8	0.51 ± 0.12	0.83 ± 0.15	3.23 ± 0.29	0.25 ± 0.04	0.26 ± 0.12	5.07 ± 0.83
40 mg/kg	8	4.12 ± 0.26 *	4.23 ± 0.26 *	5.26 ± 0.86 *	1.29 ± 0.26 *	0.72 ± 0.03 *	15.6 ± 3.25 *

* *p* < 0.05 *versus* the control group.

**Table 3:** Effect of dutasteride treatment on male reproductive indices (%).

Dutasteride	Copulation Index	Pregnancy Index	Fertility Index
0mg/kg	100 (16/16)	100 (16/16)	100 (16/16)
40mg/kg	81.3 (13/16)	38.4 (5/13) **	31.3 (5/16) *

* *p* < 0.05 and ** *p* < 0.01 versus the control group.

**Table 4:** iTRAQ analysis of differentially expressed proteins in the epididymal luminal fluids between the dutasteride group and control group.

Uniprot Accession	Gene ID	Protein Name	Gene Name	Coverage (%)	Unique Peptides	MW (kDa)	Ratio	*p*-Value
**Up-regulated proteins**								
O88752	293267	hemoglobin, epsilon 1	Hbe1	12.24	1	16.09	3.010	0.000002
P04631	25742	S100 calcium binding protein B	S100b	7.61	1	10.73	2.349	0.0002
O88205	360445	cysteine-rich secretory protein 2	Crisp2	4.12	1	27.33	1.975	0.003
Q6ZYN8	303692	α-2,6-sialyltransferase 2	St6galnac2	2.41	1	41.87	1.77	0.014
O35244	94167	peroxiredoxin 6	Prdx6	40.63	9	24.80	1.746	0.017
Q6B345	445415	S100 calcium binding protein A11 (calizzarin)	S100a11	9.18	1	11.06	1.744	0.017
Q6IE49	408234	serine peptidase inhibitor, Kazal type 2	Spink2	27.91	3	9.69	1.744	0.017
P30710	113919	glutathione peroxidase 5	Gpx5	34.84	6	25.37	1.730	0.019
P15429	25438	enolase 3, beta, muscle	Eno3	13.82	1	46.98	1.718	0.020
P14480	24366	fibrinogen beta chain	Fgb	23.38	11	54.20	1.717	0.020
P25809	29593	creatine kinase, mitochondrial 1, ubiquitous	Ckmt1b	3.83	1	47.00	1.700	0.023
P01041	25308	cystatin B (stefin B)	Cstb	7.14	1	11.19	1.634	0.036
P63255	691657	cysteine-rich intestinal protein	Crip1	23.38	3	8.54	1.631	0.037
P81827	619560	urinary protein 2	Rup2	10.89	1	10.95	1.608	0.043
Q9WUH4	25177	four and a half LIM domains 1	Fhl1	5.71	1	31.88	1.593	0.047
P37361	117038	metallothionein 3	Mt3	18.18	1	6.80	1.585	0.049
**Down-regulated proteins**								
P10860	24399	glutamate dehydrogenase 1	Glud1	1.43	1	61.38	0.667	0.014
Q32ZF7	641646	defensin beta 39	Defb39	20.55	1	8.30	0.660	0.011
Q5PQU1	24903	kininogen 1	Kng1	24.65	5	47.73	0.655	0.011
P04797	24383	glyceraldehyde-3-phosphate dehydrogenase	Gapdh	31.23	4	35.81	0.557	0.0003
Q32ZH4	641655	defensin beta 18	Defb18	10.59	1	9.92	0.532	0.0002
Q32ZI5	641631	defensin beta 2	Defb2	9.86	1	8.11	0.517	0.00007
P08932	288001	kininogen 1-like 1	Kng1l1	27.21	5	47.67	0.487	0.00001

## Abbreviations

The following abbreviations are used in this manuscript:

iTRAQ: isobaric tags for relative and absolute quantitation
DHT: dihydrotestosterone
T: testosterone
CASA: computer-assisted sperm analysis
